# Direct Reprogramming of Resident Non-Myocyte Cells and Its Potential for In Vivo Cardiac Regeneration

**DOI:** 10.3390/cells12081166

**Published:** 2023-04-15

**Authors:** Sadia Perveen, Roberto Vanni, Marco Lo Iacono, Raffaella Rastaldo, Claudia Giachino

**Affiliations:** Department of Clinical and Biological Sciences, University of Turin, 10043 Orbassano, Italy

**Keywords:** direct reprogramming, cardiac regeneration, non-myocytic cells, cardiac fibroblasts, induced cardiomyocytes, induced cardiac progenitor cells, pericytes, cardiac injury, cardiac repair

## Abstract

Cardiac diseases are the foremost cause of morbidity and mortality worldwide. The heart has limited regenerative potential; therefore, lost cardiac tissue cannot be replenished after cardiac injury. Conventional therapies are unable to restore functional cardiac tissue. In recent decades, much attention has been paid to regenerative medicine to overcome this issue. Direct reprogramming is a promising therapeutic approach in regenerative cardiac medicine that has the potential to provide in situ cardiac regeneration. It consists of direct cell fate conversion of one cell type into another, avoiding transition through an intermediary pluripotent state. In injured cardiac tissue, this strategy directs transdifferentiation of resident non-myocyte cells (NMCs) into mature functional cardiac cells that help to restore the native tissue. Over the years, developments in reprogramming methods have suggested that regulation of several intrinsic factors in NMCs can help to achieve in situ direct cardiac reprogramming. Among NMCs, endogenous cardiac fibroblasts have been studied for their potential to be directly reprogrammed into both induced cardiomyocytes and induced cardiac progenitor cells, while pericytes can transdifferentiate towards endothelial cells and smooth muscle cells. This strategy has been indicated to improve heart function and reduce fibrosis after cardiac injury in preclinical models. This review summarizes the recent updates and progress in direct cardiac reprogramming of resident NMCs for in situ cardiac regeneration.

## 1. Introduction

The heart is composed of heterogenous cellular populations that tightly regulate its function. In a mammalian heart, cardiomyocytes (CMs) occupy 70% to 85% of the volume, and non-myocyte cells (NMCs) occupy the remainder [1]. In terms of cell percentages, however, CMs are approximately 30%, whereas the more numerous, because of their small dimension, are NMCs. The latter are very heterogeneous, including cardiac fibroblasts (CFs), vascular endothelial cells (ECs), pericytes (PCs), smooth muscle cells (SMCs), macrophages, and resident cardiac progenitor cells (CPCs) [2,3]. CMs are terminally differentiated cells with very poor potential for cell renewal. Thus, in an adult heart, loss of CMs is essentially irreversible after damage, and current therapies are helpless to restore cardiac tissue. In fact, in young adult subjects, only 1% of CMs turn over annually, but this percentage is further reduced in the elderly [4,5,6]. When CM death occurs due to cardiac injury, activated fibroblasts form fibrotic tissue that replaces the damaged tissue, thus resulting in scar formation. In addition to CM loss with consequent impairment of cardiac contractility and ejection fraction, scarring negatively affects cardiac function due to the stiffness of the fibrotic scar [7]. Furthermore, due to the disruption of electrical signal conduction, arrhythmias and consequent rhythm disorders can develop. Often, after myocardial infarction (MI), these electrical decompensations are observable and are associated with a poor prognosis [8]. The available treatments in clinical practice to cope with cardiac damage include pharmacological, interventional, and surgical approaches, which, however, do not replace the lost cardiac cells. For this reason, these approaches just slow down the progression to heart failure development, providing only relief of symptoms. For complete heart failure, heart transplant is the ultimate solution in clinical practice, but it faces limitations of organ availability, possible rejection, and costs [9]. Therefore, in recent decades cardiac regeneration and reprogramming has gained significant attention.

Generation of CMs has been revolutionized by advancement in induced pluripotent stem cell (iPSC) methodologies and techniques. Although iPSC technology is currently unable to regenerate all cell types for specific customized cell therapies, it has indeed paved the path for providing tailored cells for disease modelling and drug screening [10]. In cardiovascular regenerative medicine, iPSC technology has enabled to provide CMs [11,12], but with the current differentiation methods they are mostly immature and heterogenous, creating related problems such as arrhythmogenicity, immunogenicity, and poor engraftment. Direct transplant of pluripotent stem cell (PSC)-derived CMs into the infarcted myocardium has shown limited therapeutic benefit in large animal models, mainly due to insufficient retention potential of transplanted cells for the long term, low engraftment rate, potential immunological reactions, risk of tumour induction, and inadequate electrical and mechanical coupling [13,14]. In nonhuman primates, significant retention, survival, and revascularization has been observed, but it was made possible only when one billion PSC-derived CMs were used [15]. It is expected that, for human application, even larger number of cells would be needed, which might represent a hurdle in clinical practices for this approach [16]. Recent efforts have enhanced our understanding of cardiovascular developmental biology, which may help to implement novel differentiation strategies, but only worldwide clinical trials anticipated in the coming years will help with understanding whether these multiple limitations might be overcome [17].

In this scenario, cardiac direct reprogramming has emerged as a potential solution to some of these limitations. The present review will focus on the recent updates and the potential of cardiac direct reprogramming of NMCs to restore and/or minimize the cardiac damage caused by MI.

## 2. The Discovery of Direct Reprogramming

Direct reprogramming, also referred to as transdifferentiation, is direct lineage conversion of somatic cells from one specialized cell type into another without transitioning via an intermediate pluripotent state, thus driving them towards a desired cell fate. This process needs sufficient numbers of starting cells amenable to transdifferentiate into terminally differentiated cells. Back in 1987, Davis et al. demonstrated, for the first time, that the overexpression of one transcription factor could rewrite cell fate in vitro [18]. Indeed, their finding on the possibility to convert mouse embryonic fibroblasts into myoblasts through the overexpression of a skeletal muscle cell-specific transcription factor, MYOD, prompted the revaluation of the concept of cell plasticity. After this first report, other transcription factors, either alone or in combination, demonstrated the ability to transdifferentiate one cell type into another [19,20,21,22]. These cell fate conversions, however, all took place between cells gained from the same embryonic germ layer. The milestone achieved in 2006 with the discovery of iPSCs [23] later inspired many experimental approaches that induced cell fate conversion between cells from the same or even different germ layers, leading Ieda et al. in 2010 to identify reprogramming factors that could reprogramme mouse CFs to CM-like cells in vitro [24].

Beyond in vitro approaches, in vivo cardiac direct reprogramming of NMCs offers unique advantages such as more rapid conversion by skipping the complex ex vivo isolation step, as well as selection and expansion, and it will potentially increase the differentiation efficiency, due to the interaction with mechanical and biochemical properties of the microenvironment as well as biomolecules released by ECM [25,26]. Moreover, it allows to overcome efficiently the obstacle of limited survival and integration of transplanted cells as they are resident cells, as well as to decrease tumorigenic hurdles and avoid the need of immunosuppression [11,27,28,29,30,31,32]. For these reasons, the option of in vivo cardiac direct reprogramming is emerging as a promising cardiac repair approach. In recent years, many studies allowed scientific advancement in order to decipher the molecular mechanisms underlying cardiac reprogramming [25], thus allowing the screening for candidate factors.

## 3. CF Reprogramming into Induced CMs

### 3.1. Physiological Role of CFs

In the heart, CFs are found in the interstitium and are primarily responsible for deposition of the extracellular matrix (ECM) that provides support to the contracting myocardium. CFs surround myocytes throughout the heart, where they majorly coordinate cellular and acellular turnover of connective tissues, thus maintaining the structural and cellular integrity of the heart. The heart’s collagen proteins are produced by CFs; the most abundant (85%) isoform is type I collagen, which is responsible for the strength of the heart by forming thick fibres in ECM scaffolding [33], while about 10% is represented by type III collagen, which makes thin fibres to uphold the elasticity of the the ECM [34]. The collagen-rich ECM provides structural stability to the myocardium and also encourages contractility of the cardiac tissue [35]. Besides maintaining ECM integrity, CFs also determine cell–cell and cell–matrix interactions via mechanical, electrical, and chemical stimuli [36].

Upon cardiac injury, CFs become activated and secrete proinflammatory factors, re-enter the cell cycle to promote cell proliferation, and migrate to injured areas for promoting wound healing and scar formation [37,38]. Activated CFs differentiate into myofibroblasts (MFs) that home to the injury site and produce ECM proteins, forming type I collagen-rich scars that strengthen the structure of the injured heart [39,40]. However, this stiffening of the myocardial matrix hinders contractility and promotes abnormal conduction as well as adverse left ventricular remodelling [6,41,42,43].

Cellular reprogramming ideally requires high numbers of target cells that have the capability to differentiate. Activated CFs, due to their proliferative potential following cardiac injury, represent a good source for in vivo cardiac reprogramming. Transdifferentiation of activated CFs into induced CMs (iCMs) might potentially minimize infarct thickness and scar size, hence improving cardiac function after myocardial injury. For this reason, to direct activated fibroblasts towards iCMs has emerged as an attractive option. Zhou et al. attempted to decipher the differences between beating iPSC-CMs and iCMs, both generated by transcriptome studies from CFs of the same origin. Both cell types showed CM-like molecular signatures, but iPSC-CMs expressed comparatively hyperdynamic epigenetics similar to embryonic CMs, whereas iCMs exhibited more a mature status that resembles adult CMs. In addition, in terms of metabolism, iPSC-CMs mainly employed glycolysis, whereas iCMs used fatty acid oxidation as the key pathway. Notably, iPSC-CMs and iCMs exhibited different cell-cycle status, as the cell cycle was active in iPS-CMs but inactive in iCMs [44]. Hence, directly reprogrammed iCMs are apparently more prone to acquire adult CMs-like features at the transcription level.

**Figure 1:**
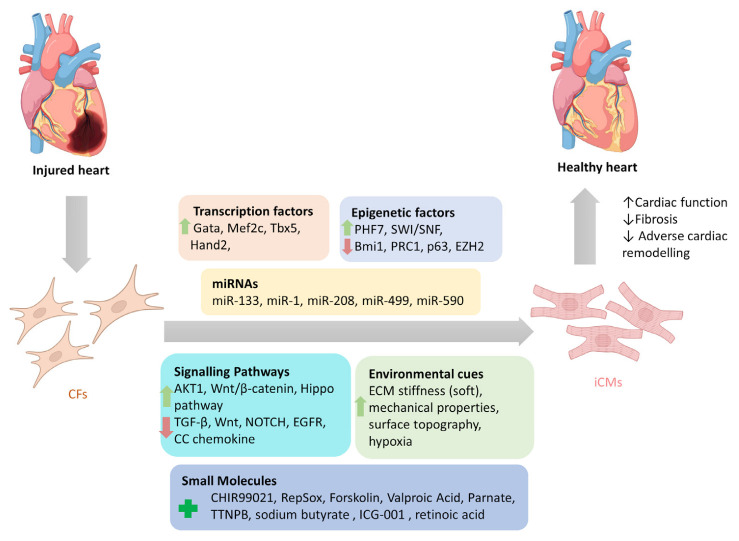
Reprogramming factors involved in direct cardiac reprogramming of cardiac fibroblasts into induced cardiomyocytes. Modulation of several reprogramming factors (separately or combined) that include transcription factors, epigenetic factors, miRNAs, signalling pathways, and environmental cues can directly reprogram cardiac fibroblasts into induced cardiomyocytes. (CFs: cardiac fibroblasts; iCMs: induced cardiomyocytes).

Several in vitro and in vivo studies have investigated how regulation of various factors in CFs can direct their reprogramming into iCMs, which will be further discussed in this section (Figure 1).

### 3.2. Transcription Factor Modulations

In fibroblasts, right expression of specific transcription factors (TFs) directs them to new cell identity. One of the direct cardiac reprogramming strategies exploits some specific genes that are expressed in the early stages of heart development. Induction of three cardiac transcription factors (TFs) expression in CFs, i.e., Gata4 (G), Mef2c (M), and Tbx5 (T), also called GMT, enabled their reprogramming into iCMs [24]. This could be exploited in the infarcted myocardium to improve heart function after cardiac injury. Inagawa et al. used a retroviral delivery approach to locally express GMT into the infarcted hearts of mice with consequent in vivo reprogramming of resident CFs into iCMs, which resulted in improved cardiac function and reduced fibrosis after MI [29]. Working along the same lines, Qian et al. confirmed that transfer of retroviral GMT in murine MI models improved cardiac function. Furthermore, when a pro-angiogenic and fibroblast-activating peptide, thymosin β4 (Tβ4), was delivered along with GMT, it resulted in further improvement of scar size and cardiac function [45]. Song et al. reported a retroviral delivery approach for the same GMT factors along with HAND2 (GHMT) to reprogram mouse CFs into CM-like cells in both in vitro and in vivo MI models, where it reduced adverse remodelling [27].

More recently, Mathison and colleagues tried to enhance CFs’ reprogramming efficacy of GMT treatment via EC transdifferentiation [31]. They showed that ECs are reprogrammed into iCMs by lentivirus encoding GMT with higher effectiveness than CFs. However, the infarcted cardiac environment is characterized by low EC and high CF cells. For this reason, before treating CFs with GMT, they transdifferentiated rat CFs into ECs using lentivirus encoding the vascular endothelial cell master regulator ETS variant 2 (ETV2). After EC markers’ expression, ETV2+GMT treatment generated a higher percentage of iCMs compared to CFs treated with the GMT cocktail alone (45% vs. 18%), while spontaneous beating was only seen in some rat iCMs obtained with ETV2+GMT treatment. Interestingly, the same group verified that this strategy could also be successfully applied to human CFs. CFs overexpressing ETV2 acquired plasticity, making them more susceptible to reprogramming [31]. Recently, it has also been observed that ETV2 can modulate epigenetics [46]. The precise reason that enabled ETV2 to contribute to direct cardiac reprogramming still needs to be further explored.

Before this, only chronic integrating viral (retrovirus and lentivirus) based approaches had been considered for gene integration into the host cell genome to induce reprogramming. This procedure, however, posed serious mutagenesis limitations in clinical feasibility and applicability of these viral vector assisted cardiac regeneration strategies. As transdifferentiating gene expression is required for a limited time to induce iCM generation, scientists thus started investigating non-integrated acute viral based approaches. Mathison and colleagues attempted to study non-integrating acute adenoviral vectors expressing GMT and compared them with integrating lentiviral vectors expressing GMT in infarcted rat hearts and found that both viral vectors were equally effective to transdifferentiate CFs in vivo into iCMs, allowing heart function improvement in post-infarct rat hearts [47]. Later, Yoo et al. attempted to employ a chimeric AAV mediated system for delivery of GMT (AAV-GMT) and Tβ4 (AAV-Tβ4) for in vivo cardiac reprogramming. This reprogramming cocktail employed combination therapy, by accomplishing GMT-mediated cardiac conversion and Tβ4-mediated angiogenic niche regeneration. In a mouse MI model, it was demonstrated that delivery of AAV-GMT and AAV-Tβ4 in the infarcted zone induced a synergistic response that led towards increased expression of cardiac-specific genes (i.e., Actc1, Gja1, Myh6, Ryr2, and cTnT) and decreased expression of fibrosis-specific genes (i.e., pro-collagen type I). After eight weeks, this reprogramming cocktail resulted in fibrosis and scar size reduction, together with cardiac recovery and vessel density enhancement [48]. Besides AAV vectors, non-integrating Sendai virus (SeV) vectors have been considered as delivery vehicles for reprogramming. Miyamoto et al. documented that SeV vectors expressing GMT made a more effective gene transfer than retroviral GMT in reprogramming resident CFs into iCMs in an in vivo model of infarcted mouse hearts [49]. Working with the same approach, more recently, Isomi et al. also reported that SeV vectors expressing GMT were able to infect resident CFs, but not CMs, vascular ECs, and SMCs, and reprogram them into iCMs in infarcted mouse hearts, where they reduced fibrosis and collagen I expression and improved cardiac function at 12 weeks after MI [50].

Besides viral-mediated delivery approaches, nanoparticle-mediated delivery has gained significant importance. Chang et al. employed cationic gold nanoparticles (AuNPs) conjugated with polyethylimine (PEI) to improve delivery of GMT without genomic integration. AuNP/GMT/PEI nanocomplex improved transdifferentiation of mouse CFs into iCM in vitro. Further in vivo study in an infarcted mouse model also highlighted significant success of this approach as injection of this nanocomplex showed improvement in cardiac structure and function two weeks after MI. In addition, reduced scar size and recovered thick myocyte bands were observed in the infarcted zone. In terms of cell maturation, this approach seemed more successful in vivo as indicated by the production of more mature iCMs in the heart environment compared to the in vitro condition. This was pointed out not only by the increased expression of Tbx5 but also by the augmented level of sarcomeric proteins (such as α-Actinin, cTNT, and α-MHC), which resulted in a well-organized sarcomere structure, essential for improving cardiac function, although cardiac functionality was not assessed by the authors. Most likely, the promising in vivo results relied on ECM and growth factor cues provided by the cardiac microenvironment that enabled reprogrammed iCMs to develop into a more mature phenotype [51].

Wang et al. highlighted that G, M, and T protein stoichiometry in iCM reprogramming also holds significance. They found that polycistronic vectors expressing relatively high levels of Mef2c and low levels of Gata4 and Tbx5 allowed efficient reprogramming of mouse primary CFs into iCMs by more than tenfold compared to unbalanced G, M, and T protein expression [52]. The same research laboratory further showed by genetic lineage tracing in a murine MI model that retroviruses encoding single triplet GMT produced more mature iCMs with assembled sarcomere structures in the infarcted region after four weeks of delivery compared to groups receiving viruses encoding separate G/M/T. Indeed, serial high-resolution echocardiography, performed at four and eight weeks post-MI, evidenced a significant improvement in heart function as depicted by improved ejection fraction and fractional shortening in the GMT group [32].

Recently, Tani et al. created a novel Tcf21iCre/reporter/MGTH2A transgenic mouse system for expression of Mef2c/Gata4/Tbx5/Hand2 (MGTH) to repair chronic MI in mice. It was found that this therapeutic approach has reprogrammed ≈2% of resident CFs into iCMs. Notably, a majority of iCMs were the result of bona fide cardiac reprogramming rather than fusion with CMs. Significant improvement in myocardial contraction and reduced fibrosis was observed. Microarray studies found that the MGTH overexpression activated cardiac program and simultaneously suppressed fibroblast and inflammatory signatures in chronic MI. Furthermore, single-cell RNA sequencing revealed that cardiac reprogramming stimulated conversion of profibrotic CFs to a quiescent antifibrotic state. MGHT overexpression suppressed Meox1, a key gene involved in fibroblast activation that has partly contributed to induction of antifibrotic effects. However, the precise mechanism that has contributed to the MGTH-mediated transcriptional switch between cardiac program activation and fibrotic suppression remains unexplored [53].

It soon became evident, however, that for cardiac reprogramming, the combination of TFs appeared to be species-specific. In fact, it has been demonstrated that GMT was able to induce cardiac reprogramming in mouse fibroblasts [45], whereas the same combination was ineffective in pigs [54] and humans [55].

Overall, over the years it has been realized that the efficiency of these cocktails involving only TFs is inadequate for the reprogramming of human CFs into iCMs; for this reason, strategies that combined TFs with other factors were explored. Furthermore, for a safer clinical application, investigations are presently focusing on alternative strategies to the integrating viral vectors. Recently, significant progress on in vivo cardiac reprogramming has been made by introducing for the first time non-integrating viral vector systems to in vivo transgene-based cardiac reprogramming. Since successful target selection is needed for transferring the in vivo cardiac reprogramming procedure into a therapeutic application, promoter-based selective targeting for NMCs would be worth deep investigation in this field.

### 3.3. Epigenetic Regulation

During cardiac reprogramming in CFs, waves of chromatin remodelling events begin at the transcriptomic level and include rapid accession of cardiac gene signatures and progressive loss of CF molecular program [56,57,58,59].

This transcription remodelling is driven by upstream chromatin landscape repatterning that involves expurgation of epigenetic hurdles to reprogramming, including, for example, histone modifications and suppressive DNA methylation [60]. Epigenetic regulations have been shown to play a critical role in cardiac fate determination. Polycomb complex protein BMI-1 is bound to cardiac loci in CFs, where it suppresses the expression of cardiac genes, thus representing an epigenetic hurdle in the early stages of iCM reprogramming. Depletion of BMI-1 in mouse CFs has shown an increase in active histone mark H3K4me3 and a decrease in suppressive mark H2AK119ub, and this modulation results in improved cardiac reprogramming of CFs [61]. H3K4 methyltransferase Mll1 and factor Men1 is also a barrier in iCM reprogramming in a mouse model; in fact, inhibition of Mll1 and Men1 enhanced the efficiency of iCM formation [62]. Conversely, inhibition of EZH2, which encodes H3K27me3, a histone methyltransferase enzyme, promotes cardiac gene re-activation via removal of epigenetic repression during direct cardiac reprogramming of a human ESC-derived fibroblast line [63]. In adult human CFs, Garry et al. identified histone reader PHF7 as a key factor to overcome epigenetic barriers by localizing to cardiac super enhancers and collaborating with the SWI/SNF complex to improve chromatin accessibility in order to allow GMT binding at these enhancers. It was highlighted that PHF7 enhanced reprogramming of adult human CFs into iCMs by three- to fourfold when added to myocardin and GHMT reprogramming cocktail [64]. Identification of the prominent role of PHF7 reinforces the necessity to also focus on other epigenetic factors to fully decipher the mechanistic details of direct cardiac reprogramming.

Silencing of p63, an epigenetic regulator, has also been shown to enhance transdifferentiation of neonatal rat and adult human CFs into iCMs. Recently, Pinnamaneni and colleagues have reported p63 silencing by short hairpin RNA for p63 (shp63) along with cardiogenic differentiation factors Hand2 and Myocardin enhanced expression of CM markers in CFs compared to GMT treatment with or without shp63. Further investigation revealed that epigenetic modulations played a key role as overexpression of the p63 motif transactivation inhibitory domain (TID) inhibited p63 binding with histone deacetylase 1 (HDAC1), and p63-TID+ Hand2/Myocardin resulted in better cardiac reprogramming of CFs into iCM compared to the shp63+Hand2/Myocardin combination. It was concluded that the p63-TID overexpression reprogramming strategy could have the potential to circumvent epigenetic hurdles to CF cardio-differentiation [65].

Overall epigenetic barriers to reprogramming identified in mice and humans are different. Integrative analysis of transcriptomic and epigenomic data are providing a new mechanistic understanding of human iCM reprogramming.

### 3.4. microRNA Regulation

Beyond the previous epigenetic regulation approaches, microRNAs (miRNAs) also hold the potential for direct cardiac reprogramming. miRNAs are small RNA molecules that, based on sequence complementarity, recognize target mRNAs, resulting in their silencing.

miRNAs along with a combination of TFs and other various factors have been reported to be useful in the conversion of human CFs into CMs. Nam et al. reported that a combination of GHMT, Myocardin with miR-1, and miR-133 has the ability to transdifferentiate human adult CFs into CMs [55]. Singh et al. demonstrated that lentiviral delivery of GHMT TFs coupled with Myocardin or miR-590 produced iCMs from rat, porcine, and human CFs in vitro. miR-590 promoted suppression of Sp1, a zinc-finger protein, which upregulated numerous genes linked with CM phenotype and downregulated various fibroblast-related genes [66]. Years later, Zhou et al. reported that GMT combined with miR−133 (GMT133) has the ability to convert human CFs into CMs [57]. More recently, Tang et al. reported that when TBX20 is added to conventional reprogramming cocktail GMT133 (GMT133+TBX20), it resulted in enhanced acquisition of functional iCMs exhibiting enhanced cardiac function in contractility and mitochondrial respiration [67].

Selected miRNA clusters can also be employed for cell reprogramming without involving exogenous TFs. On the basis of the fact that a single miRNA has the ability to regulate multiple transcripts, it is considered that miRNA clusters can synergistically induce cellular reprogramming by regulating multiple targets. Jayawardena et al. found that four microRNAs (miR-1, 133a, 208, 499) termed as miRcombo combined with JAK inhibitor I treatment was ample to induce in vitro cardiac reprogramming of CFs into iCMs. They further proved it in mice through lineage-tracing methods by employing a lentiviral delivery approach to deliver miRcombo in in vivo infarcted mouse hearts [30]. Later, the same research group validated these results in an extensive in vivo study in mouse models and found that in the infarcted myocardium, NMCs can be transdifferentiated in situ into mature functional CMs that promoted cardiac regeneration after myocardial injury [68]. In another recent study, this group has shown in neonatal CFs that the addition of ICR2, an RNA-sensing receptor ligand, to miRcombo further enhanced the capability of reprogramming factors to produce iCMs by targeting the RNA-sensing receptors Rig-I and TLR3 [69]. Kang et al. suggested that optimizing miRcombo delivery at a stoichiometric ratio is necessary for efficient cellular reprogramming. For this purpose, they developed a polycistronic vector that induced equal expression of four microRNAs of miRcombo and found that this vector with an AAV delivery approach was robust in direct reprogramming of mouse CFs into CMs in mouse MI models [70].

To understand whether miRcombo can be translated into humans, recently it was shown that miRcombo has the ability to in vitro transfect adult human CFs and induce their reprogramming into iCMs [71]. Although clinical studies are required to prove this fact, this study has indicated that this cocktail might be effective in adult human CFs. Recently, Baksh et al. investigated the possibility of species-specific limitations to miRcombo. They observed, however, that in several mammalian species (dogs, pigs, humans) transfection of miRcombo worked effectively in CFs isolated from their left ventricle and induced their direct conversion into CM-like cells [72].

During this course of in vivo cardiac reprogramming, scientists have also made tremendous efforts to improve delivery methods of miRcombo in infarcted hearts, as direct administration of naked miRNAs is difficult based on their negatively charged surface and potential swift degradation. Hence, effective, biosafe, noninvasive, and biocompatible vehicles are obligatory to protect and deliver miRNAs into the targeted region. In an effort to improve the delivery method of miRcombo for in vivo reprogramming, Wang et al. loaded miRcombo on mesoporous silicon nanoparticles coated with FH peptide-modified neutrophil-mimicking membranes (sequence: FHKHKSPALSPV). They used this construct in in vivo infarcted mouse hearts and found that resident CFs were induced to reprogram into iCMs. The attenuation of fibrosis accompanied by the improvement of cardiac function prove that the use of nanotechnology is a successful strategy to deliver miRNA [73]. Other authors employed engineered nanotechnologies for improving miRNA delivery. Muniyandi and colleagues demonstrated in vitro that co-delivery of miR-133a and miR-1 using Poly (lactic-co-glycolic acid)/Polyethylenimine (PLGA-PEI) nanospheres encouraged intracellular internalization, displayed pH-mediated release of miRNA, and efficiently reprogramed adult human CFs into mature iCMs [74]. They later improved the delivery approach by using for the first time an electrospinning strategy to fabricate porous and smooth PLLA scaffolds for direct cardiac reprogramming. These scaffolds were further functionalized with fibronectin, and miR133a/miR1 PEI polyplexes were immobilized on them. Adult human CFs cultured on these scaffolds exhibited cell adhesion and proliferation, and they significantly absorbed the miRNAs. This strategy increased the loading efficiency of miR133a/miR1 and allowed their release in a pH-dependent biphasic pattern that overall helped to determine the cell fate of CFs precisely. After one week of transfection, expression of iCM signature markers confirmed that CF reprogramming took place [75]. This approach holds potential to be translated in vivo in infarcted hearts. Later, this group attempted to determine the influence of epigenetic reprogramming on cell fate determination, by hypothesizing that demethylation can enhance direct cardiac reprogramming. For this purpose, they considered the role of miR133a in epigenetic regulation and of 5′Azacytidine in inhibiting DNA methylase, and they tried to co-deliver 5′Azacytidine and miR-133a encapsulated in PLGA-PEI nanoformulations in adult human CFs. Prominent cardiac features were observed 7 days post-transfection as indicated by increased cTnT+ cells and increased expression of cardiac TFs (i.e., GATA4, MEF2C, TBX5, HAND2, and NKX2.5). This cocktail also appeared to regulate net DNA methylation, as indicated by reduced 5-methylcytosine levels. These results emphasized that co-delivery of miR133a and 5′Azacytidine dictated CF fate towards iCMs [76]. However, this study did not provide mechanistic details of the role of reduced DNA methylation in direct cardiac reprogramming.

In further studies, different nanomaterials have been employed to deliver the reprogramming cocktail. Yang et al. used branched polyethyleneimine (BP) coated nitrogen-enriched carbon dots (BP-NCDs) to load miRcombo. In mouse CFs, delivery of this nanocomplex, within three weeks, produced a significant amount of contractile iCMs, whose presence was also confirmed by increased expression of cardiac markers, i.e., Gata4, Tbx5 Hand2, NPPA, NKX2-5, MYH7, and TNNT2. Furthermore, this approach was tested injecting this nanocomplex into the epicardium along the border zone of infarcted mice, which, after 4 weeks, showed a significant decrease in cardiac fibrosis and infarct thickness [77]. Recently, Nicoletti and colleagues investigated a lipoplex based nanosystem to carry miRcombo in adult human CFs [78]. In this study, lipoplexes were composed of helper dioleoyl phosphatidylethanolamine (DOPE) and cationic lipid [2-(2,3-didodecyloxypropyl)-hydroxyethyl] ammonium bromide (DE). In an in vitro study, DE-DOPE lipoplexes with nanometric hydrodynamic size (372 nm) showed high loading proficiency (99%), quicker miRNA release (99%), and high viability (80–100%) compared to commercial lipoplexes. In addition, DE-DOPE lipoplexes increased CF reprogramming into iCM cells as indicated by increased expression of CM markers [78].

Overall, the potentiality of different miRNA cocktails for direct reprogramming is clearly emerging and might be effective in adult human cells, accompanied by recent progress to improve their delivery methods through the use of different biosafe and biocompatible nanomaterials. Further investigation in cardiac tissue mimetics and in vivo studies are necessary, however, to demonstrate the full potential to improve cardiac reprogramming and maturation in the presence of biochemical and biophysical stimuli.

### 3.5. Modulation of Intracellular Signalling Pathways

Cardiac reprogramming can be done successfully by modulating the genes that are involved in the activation of heart genetic program and maintenance of fibroblast identity. Indeed, it was proved that pro-fibrotic pathways, including TGFβ and Wnt signalling, served as barriers to heart reprogramming, and their inhibition would enhance cardiac reprogramming. Overactivation of the transforming growth factor beta (TGF-β) pathway attenuated cardiac regeneration, but its inhibition enhanced cardiac reprogramming from CFs to iCMs [79,80,81,82]. It has also been reported that TGFβ signalling inhibition and cAMP signalling activation decreased differentiation of myofibroblasts and enhanced iCM generation [81]. In fact, miR-133-mediated suppression of snail1, the downstream target of the TGFβ pathway, resulted in more production of iCMs than using GMT alone [83]. Moreover, combinatorial silencing of both Wnt and TGFβ signalling pathways enhanced the efficiency, quality, and speed of iCM production from CFs by coupling TGFβ and Wnt inhibitors with the GMT cocktail. It is important to note that even though the TGFβ pathway activates in CFs after MI, in vivo CF direct reprogramming produces more fully reprogrammed iCMs than their in vitro cultured counterparts [84].

Kaur et al. used a modified mRNA (modRNA) reprogramming cocktail, named 7G-modRNA, comprising GHMT, along with an inhibitor of TGFβ (DN-TGFβ), an inhibitor of Wnt (DN-Wnt8a), and acid ceramidase (AC) to reprogram NMCs into iCMs during ischemic conditions after MI in mouse models. It was found that pro-angiogenic factors were upregulated, whereas fibroblast markers were downregulated. This cocktail reprogrammed CM-like cells in the scar area, reduced scar size, promoted capillary density, improved cardiac function, and increased survival after MI. However, it was unable to develop de novo beating CMs either in vitro or in vivo [85]. This limitation of modRNA might be due to the relatively short transgene expression time that was insufficient to reprogram non-CMs into mature CMs.

NOTCH signalling plays an important role during development and maturation of CMs. Activated NOTCH signalling promotes differentiation of NMCs into the cardiogenic lineage but prevents CM differentiation. Therefore, in developing myocardium inhibition of the NOTCH pathway is important to obtain fully mature and functional CMs [86]. Downregulation of the NOTCH pathway has shown to improve GHMT-mediated reprogramming efficiency of mouse embryonic and tail tip fibroblasts into iCMs [87]. Conversely, another recent in vivo study in mice has reported that the NOTCH1 pathway was activated in the NMCs in the stressed heart. Inhibition of the NOTCH1 pathway and knockdown of Wisper, a long non-coding RNA in CFs that regulates fibrosis, resulted in limited fibrosis and stimulated formation of CMs from NMCs [88]. In addition to pro-fibrotic pathways, recent research has also determined the impact of other intracellular signalling on heart reprogramming. Zhou et al. performed screening of a protein library and determined that iCM conversion and maturation starting from CFs could be enhanced by the activation of the Akt/protein kinase B pathway along with GHMT. In the GHMT cocktail, addition of Akt1 induced mature iCMs as indicated by increased polynucleation, cellular hypertrophy, mature cardiac gene levels, and metabolic reprogramming. Further mechanistic details indicated that IGF1 and PI3K were active upstream of Akt, whereas mTORC1 and Foxo3a were involved downstream of Akt1 signalling to propel CFs to iCM reprogramming. It was also noted that, in comparison to adult tail tip fibroblasts, adult CFs were more willing to reprogram into iCMs because GHMT expression in CFs remained low, although active; therefore, it was insufficient to turn on their reprogramming into iCMs [89]. The same research group further attempted to decipher the genomic targets and interactions by genome-wide analyses and enhancer profiling during cardiac reprogramming of CFs into iCMs employing the same Akt/GHMT cocktail. It was found that TFs played a synergistic role to trigger enhancer activation mainly enriched with Mef2c binding sites. During the course of reprogramming, Hand2 and Akt1 coordinated to activate additional cardiac enhancer elements in a sequential manner that helped to recruit TFs to these sites according to temporal acquisition of functional phenotypes in iCMs. This overall resulted in augmented cardiac genes expression. Interestingly, these enhancer landscapes together imitated enhancer activation pattern during embryonic cardiogenesis. Performing a study on gene regulatory network, these authors highlighted that suppression of genes involved in EGFR signalling might occur by ectopic binding of these reprogramming TFs. This evidence was confirmed by chemical inhibition of EGFR signalling that resulted in increased cardiac reprogramming [90].

Besides the previously presented pathways, various other signalling pathways can be regulated to enhance and improve cardiac reprogramming. Wang et al. identified that autophagy is also linked with direct cardiac reprogramming. In MGT-mediated direct cardiac reprogramming, autophagy inhibition by downregulation of autophagic factor Beclin1 (Becn1) activated Wnt/β-catenin signalling, which encouraged iCM induction and maturation. Infarcted mouse models with Becn1 haploinsufficiency also showed improved cardiac reprogramming mediated recovery as indicated by decreased scar size and improved cardiac function [91]. Working to identify and target different signalling pathways for cardiac reprogramming, Singh et al. found that the Hippo pathway intermediate Tead1 plays an important role in cardiac reprogramming as it increased the transdifferentiation of CFs into mature iCMs. They observed that in comparison with the standard GMT cocktail, Hippo pathway effector Tead1 in conjunction with Gata4 and Mef2c enhanced reprogramming of rat and human CFs into iCMs; hence, this reprogramming cocktail can have further potential application for in vivo cardiac regeneration [92]. The importance of modulating inflammatory pathways for reprogramming has also been considered. Gu et al. reported the ability of an IMAP cocktail comprising four molecules, insulin-like growth factor-1 (IGF1) (I), an inhibitor of Mll1 (M–MM589), an inhibitor of TGF-β (A–A83-01), and PTC-209 (P), to augment the efficiency of GMT-driven cardiac reprogramming through the specific suppression of C-C chemokine signalling pathways [93]. This finding indicated that suppression of inflammatory pathways is capable of enhancing the transdifferentiation capability of neonatal CFs into CMs, which displayed a higher maturity as evidenced by spontaneous increase in beating and calcium transient.

Overall, this set of scientific studies recognizes major signalling pathway involvement in cardiac reprogramming. This offers the potential to consider a targeted modulation of these signalling pathways for improving therapeutic outputs in in vivo direct cardiac reprogramming.

### 3.6. Environmental Cues

The local cardiac environment has an important impact on CF direct reprogramming into CMs for cardiac regeneration [25]. Environmental cues such as ECM components also affect reprogramming efficiency. Substrate mechanical properties such as its stiffness can influence the differentiation and maturation of iCMs. As far as the role of mechano-transduction and matrix stiffness on cardiac reprogramming is concerned, Kurotsu et al. found that a soft hydrogel substrate could improve the quality and efficiency of CF reprogramming into CMs through the YAP/TAZ signalling pathway and suppressed CF programs [94]. CFs culture on microgrooved substrates enhanced the efficiency of generated CMs and the organization of their sarcomeric structures as well. These kinds of surface modifications induce a permissive environment by expression of pro-reprogramming modulators, which include myocardin sumoylation (post-translational modification), MKI1 (mechanosensitive transcription factor), and histone H3 acetylation (chromatin remodelling) [95]. Li et al. compared the effect of 2D culture vs. 3D culture on miRcombo-mediated direct reprogramming of neonatal CFs into iCM. They found that the fibrin-based 3D tissue-engineered environment better mimicked native cardiac tissue and enhanced direct reprogramming of neonatal CFs into CMs. This was linked with increased expression of matrix metalloproteinases (MMPs) in the 3D culture. They further confirmed this by employing a MMP inhibitor that retracted the effect of the 3D culture on miRcombo reprogramming [96]. In infarcted hearts, MMP levels are upregulated [97], which can also be a reason why in vivo direct cardiac reprogramming can have more successful outcomes compared to in vitro reprogramming.

Furthermore, the iCMs generated from in vivo reprogramming of CFs are considered more mature at functional and transcriptional levels than those produced in vitro. This might be due to the presence of in vivo mechanical and biochemical signals. The myocardium environment is dynamic; cells are contiguous with one another and are facing the continuous contractile forces in a specific direction. Moreover, CFs are exposed to several chemokines and growth factors that might enhance the yield of reprogramming. Van et al. pointed out that after injury a particular subpopulation of CFs was directly programmed into iCMs, and this process was further stimulated by local mechanical properties and topographical cues of the microenvironment [98]. The importance of cardiac tissue-like biochemical and biophysical stimuli on direct reprogramming was confirmed by Paoletti and colleagues, who found that in a 3D microenvironment that mimicked cardiac tissue, miRcombo-based reprogramming cocktail efficiency was enhanced on human CFs [99].

Overall, both in vitro and in vivo direct reprogramming approaches can benefit from the cues offered by the local cardiac environment. The superior results obtained in vitro in the presence of materials mimicking cardiac stiffness and/or microstructure, as well as the promising results achieved with in situ approaches, indicate the pivotal role of the permissive cardiac environment in directing a more complete target cell transdifferentiation.

### 3.7. Small-Molecule-Based Transgene-Free Strategies

In spite of the relatively high transfection efficiency of viral protocols, concerns related to insertional mutagenesis, residual expression, and genetic/epigenetic aberrations still exist that impose serious constraints to their use in clinical practice [100,101,102]. For this reason, small molecule–driven, non-viral, and transgene-free strategies (including the previously presented miRNA cocktails) have gained appraisal in terms to improve the safety profiles of reprogramming protocols [25,103].

A pure chemical cocktail was demonstrated capable to functionally substitute the ectopic expression of TFs in inducing beating clusters of CMs from mouse CFs, both in vitro and in vivo, although with low efficiency [104,105]. This cocktail was named CRFVPT and comprised six chemicals: a GSK3 inhibitor (C–CHIR99021), a TGFbR1 inhibitor (R–RepSox), a molecule sustaining cAMP synthesis (F–Forskolin), a HDAC inhibitor (V–Valproic Acid), an inhibitor of lysine-specific demethylase 1 (P–Parnate), and a selective analogue of retinoic acid (T–TTNPB). Although its precise mechanism of action remained not perfectly clarified, CHIR99021 and RepSox, the two ‘mesenchymal to epithelial transition’ modulators, can be argued to suppress the fibroblast phenotype. In contrast, Parnate and VPA, the two epigenetic modulators, can be argued to help clear the epigenetic obstacles found in different cell types. The final two factors are somehow capable of inducing the features of the CM-like cells. An enriched chemical cocktail composed of nine molecules (CHIR99021, A83-01, SC1, OAC2, Y27632, BIX01294, AS8351, SU16F, JNJ10198409), partially overlapping with the one used to reprogram mouse fibroblasts, was also reported to reprogram human fibroblasts in beating cardiac cell clusters [106]. More recently, Testa and colleagues demonstrated that CRFVPT cocktail efficacy could be enhanced by pharmacological inhibition of the epigenetic modulator Bmi1, resulting in repression of the JAK/STAT3 and MAPK/ERK1/2 pathways in iCMs generated from mouse CFs. This resulted in increased iCM spontaneous beating and high levels of mature cardiac phenotype markers MLC-2v and cTNT [107]. These data also sustain the importance of inflammatory pathway repression to improve CF transdifferentiation efficiency.

Besides the use of pure chemical cocktails, Singh et al. demonstrated that addition of small molecules in conventional TF cocktails can enhance generation of iCMs and smash the barriers to cardiac reprogramming. The addition of three small molecules, i.e., sodium butyrate (histone deacetylase inhibitor), ICG-001 (WNT inhibitor), and retinoic acid (cardiac growth regulator), in the conventional GMT cocktail enhanced direct reprogramming of adult rat and human CFs into iCMs compared to the efficiency of small molecules or GMT alone or the GHMT/myocardin+miR-590 cocktail [108]. This study suggested that besides this combination, various other small molecules along with intracellular factors can be employed and optimised to enhance reprogramming outcomes. This will be beneficial in suggesting opportunities for improvement of translational strategies for in vivo cardiac regeneration.

Overall, these studies open the way to the ultimate aim to regenerate cardiac tissue in diseased hearts using only druggable molecules. While the chemical in vivo cardiac reprogramming approach is an interesting chance for achieving clinically feasible therapeutic interventions, however, a critical matter still facing the chemical approach is the selective targeting of cardiac cells or CFs for iCM generation without impacting other organs’ cells, thus avoiding unwanted side effects. In addition, chemical reprogramming requires deeper knowledge of the protocols to adopt (such as dose and duration of the administration of factors/chemicals, routes of delivery, etc.).

## 4. CF Reprogramming into Induced CPCs

Numerous studies have investigated CF reprogramming into induced CPCs (iCPCs) which display the potential capability to differentiate into three cardiovascular lineages that include ECs, SMCs, and CMs [109]. Upon transplantation in an injured heart, iCPCs may contribute to numerous processes including neovascularization and favourable remodelling of the cardiac scar. In this regard, Lalit and colleagues attempted to improve the in vitro reprogramming efficiency of CFs into iCPCs [110]. They tested 22 genes individually cloned into a doxycycline (dox) inducible lentiviral vector as candidates for reprogramming the capability of mouse CFs into iCPCs. They showed that five (MTGNB; Mesp1, Tbx5, Gata4, Nkx2.5, and Baf60c) cardiac TFs when coupled with small molecules, i.e., *BIO* 6-bromoindirubin-30-oxime (a Wnt activator) and *LIF* leukaemia inhibitory factor (a JAK/STAT activator), could generate iCPCs. These resulting cells were expandable and capable of self-renewal, thus overcoming the low efficiency of the reprogramming technique. This research also showed that iCPCs differentiated preferentially into iCMs (about 80–90%), while the percentage of SMCs and ECs could be increased when cultured in the presence of vascular differentiation conditions. However, these vascular components were not found in an in vivo model [110]. Even if iCPC-derived CMs displayed organized sarcomeres, they were not capable of spontaneous contraction; they became more mature after co-culture only [110]. Moreover, these authors evidenced that the activation of the Wnt and JAK/STAT pathways had the potential to reprogram CFs into Flk1+, PDGFRα+, and Isl1+ iCPCs and stabilize them. Also, these mesoderm-limited progenitors displayed the capability to differentiate into CMs, ECs, and SMCs in vivo when injected into post-MI mouse hearts [110]. The same research laboratory in another study attempted to generate functional cardiac tissue from iCPCs obtained through reprogramming of adult mouse CFs by employing the same reprogramming cocktail (MTGNB) together with activation of canonical Wnt and JAK/STAT signalling. They found that iCPCs were able to expand, repopulate, and differentiate into ECs, SMCs, and CMs and formed electrically coupled cardiac tissue in decellularized 3D native cardiac ECM scaffolds. However, the reprogramming efficiency was very low [111].

Interestingly, Zhang et al. identified other signalling molecules (BMPAW: such as BMP, Activin A, and Wnt) that allowed successful generation of reprogrammed Flk1+, PDGFRα+, Isl1+, Nkx2-5+ iCPCs from embryonic fibroblasts, which could differentiate in the three main cardiac lineages with a very high efficiency. When transplanted into an infarcted mouse heart, they induced moderate neovascularization, a significant smaller infarct size, and cardiac performance recovery, limiting the long-term (3 months) adverse remodelling. Notably, it was also evidenced that more than 90% of the iCPCs differentiated into cardiac lineage; in particular, the authors estimated the conversion markers, i.e., 59% of SMCs, 31% of CMs, and only 7% of ECs [112]. The successful expansion was initially restricted to murine iCPCs only; however, this limitation has been recently overcome by Wang and co-workers (2022). Although not starting from resident NMCs, these authors showed in vitro that human foreskin fibroblasts could be reprogrammed into iCPCs using six small molecules (CHIR99021, A83-01, GSK126, Forskolin (an adenylyl cyclase activator), CTPB (a P300 histone acetyl transferase activator), and AM580 (a RARα activator). These human iCPCs possessed long-term self-renewing and expandable properties with preservation of the CPC phenotype and differentiation capacity towards cardiac lineages. When transplanted into an infarcted mouse heart, iCPCs improved cardiac function up to 13 weeks [113]. It will be interesting to assess whether similar results might also be obtained starting from human CFs. Yu and colleagues reported a very interesting study that demonstrated that mouse CFs can be chemically induced into cardiac progenitors using TGF-β/Alk5 inhibition (by SB431542 or RepSox/AZ12799734 inhibitors) coupled with hypoxia [114]. This reprogramming strategy generated two progenitor populations of different potency. Early in the reprogramming process, a first population appeared that comprised ECs and CMs, whereas further sustained culture in the same reprogramming media gave rise to a second population that was directed towards smooth muscle lineages and eventually formed SMCs. Further characterization using a CRISPR-knockout screening approach helped to recognize the contribution of DNA methyltransferase 1-associated protein 1 (Dmap1) in regulating this reprogramming. Dmap1 loss decreased promoter methylation, enhanced Nkx2.5 expression, and encouraged self-renewal, although sustained cadherin-1 expression repressed further differentiation [114]. Additional optimization of this protocol can help to simultaneously preserve all three cell types and their self-renewal. This study paved the path for the employment of this approach for in vivo induction of cardiac progenitors that can potentially enhance cardiac function. Further studies are required to identify the optimised combination of the chemical cocktail in order to improve reprogramming effectiveness and the appropriate balance among the three cardiac cell components.

**Figure 2:**
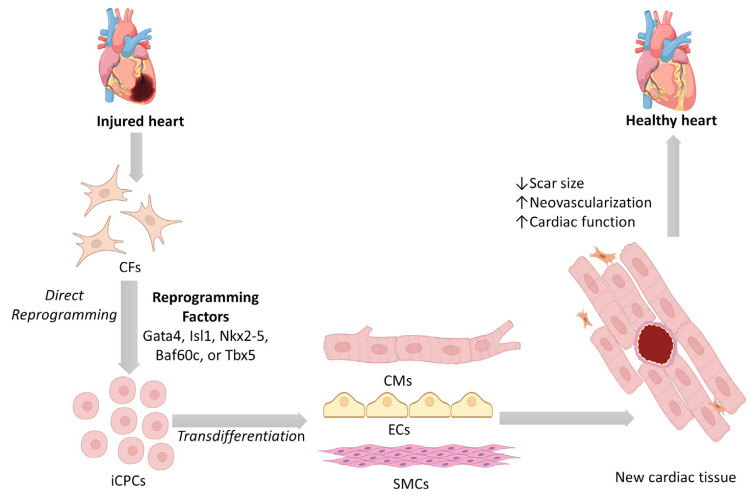
Direct reprogramming of cardiac fibroblasts into induced cardiac progenitor cells and their potential role for repairing damaged heart. Cardiac fibroblasts can be reprogrammed into induced cardiac progenitor cells. Both resident and induced cardiac progenitor cells can be transdifferentiated into three cardiac lineages, i.e., cardiomyocytes, endothelial cells, and smooth muscle cells. This can potentially result in reducing cardiac scar size, promoting neovascularization, and improving cardiac function. (CFs: cardiac fibroblasts; iCPCs: induced cardiac progenitor cells; CMs: cardiomyocytes; ECs: endothelial cells; SMCs: smooth muscle cells).

In vivo direct reprogramming of resident CFs into iCPCs via injection of the reprogramming cocktail directly into the damaged cardiac tissue can potentially give rise to all three cardiac cell lineages (Figure 2). The advantage of this approach consists of avoiding transplanted cell engraftment issues (poor cell retention, survival, and engraftment) in the injured cardiac environment and all the steps for cell extraction, reprogramming, and expansion [115]. Moreover, Lalit et al. indicated as a limitation of the in vitro remodelling that fibroblast culture can contain karyotypically abnormal cells with the consequent high probability of chromosomal abnormalities in iCPCs [116].

Recently, Liang and Wang have reported their attempts to in vivo reprogram mouse CFs into iCPCs in infarcted hearts by using a CRISPR approach. The reprogramming cocktail, containing a novel fibroblast-specific CRISPR model (Col1a2-cre/ERT; CAG-cas9) and single guide RNAs (sgRNAs) to target the core promoter region of Gata4, Isl1, Nkx2-5, Baf60c, or Tbx5, was introduced locally through an AAV system in infarcted mouse hearts. It was observed that the CRISPR system was activated in approximately 50% of CFs after tamoxifen induction. In addition, it was also observed that iCPCs had subsequently differentiated into cTnT+ CMs, α-SMA+ SMCs, or CD31+ ECs. Ejection fraction and scar formation were improved in these infarcted mouse hearts. It was concluded that endogenous loci can be activated in vivo to reprogram CFs into iCPCs that can further differentiate into mature cardiovascular cells [117]. This study has paved the path for cardiac regenerative medicine based on in vivo obtainment of iCPCs.

Notably, however, protocols leading to reprogrammed iCPCs starting from murine CFs are still to be adapted for human cells. Overall, since it is well known that cell expansion is a fundamental key in regenerative medicine, the capability of transdifferentiating the numerous CFs that become activated and highly proliferate after myocardial damage into iCMs or, even better, into iCPCs with their intrinsic self-renewal potential and multi-lineage differentiation ability is of outmost interest in the field of cardiac regeneration. Indeed, if successful, this approach would open a novel perspective for cardiac failure treatment by redirecting the activity of pro-fibrotic cells into a regeneration process of functional cardiac cells.

## 5. PC Reprogramming into Induced Vascular SMCs

### 5.1. PCs in the Heart: Physiological Role

PCs are mesoderm-derived cells, one of the abundant but most unknowable and vaguely defined cell population in the heart [118]. They are mural cells that wrap around ECs in microvessels such as capillaries, terminal arterioles, and precapillary venules. Notably, they are particularly abundant in the myocardial capillaries with respect to other organs. In addition to structural support of the ECs, they also exert different functions such as vessel stabilization and angiogenesis, vascular remodelling, capillary blood flow control, and vascular permeability regulation. PCs are positive for numerous markers, depending on their stage and location, but none of them is capable of definitively identifying them. Furthermore, PCs and MFs share some antigenic markers (e.g., α-SMA and PDGFR-β) but are assumed to have definite functional tasks in vascular remodelling, vascular stability, and protracted contractions after ischemia-reperfusion injury [119,120,121]. However, the lack of unambiguous markers has so far impeded complete comprehension of PC plasticity in homeostasis and regeneration. In the developing murine heart, a lineage tracing approach has shown that epicardial PCs are descendants of vascular SMCs [122].

Vascular architecture in the infarcted cardiac region is disrupted, promoting vascular disintegration and capillary rarefaction [123]. The outcome is gradual decrease in the blood supply, which hinders oxygen supply, nutrients, and elimination of metabolic waste, promoting CM necrosis. Therefore, for cardiac regeneration in addition to functional CMs, reconstruction of vascular architecture is also essential to restore cardiac function [124,125]. Hence, therapeutic approaches that can promote endogenous vascularization in situ are noteworthy. In this context, PCs might represent an interesting option for this purpose under the hypothesis that PC reprogramming can promote neovascularization.

### 5.2. PC Reprogramming Strategies to Obtain Induced SMCs

**Figure 3:**
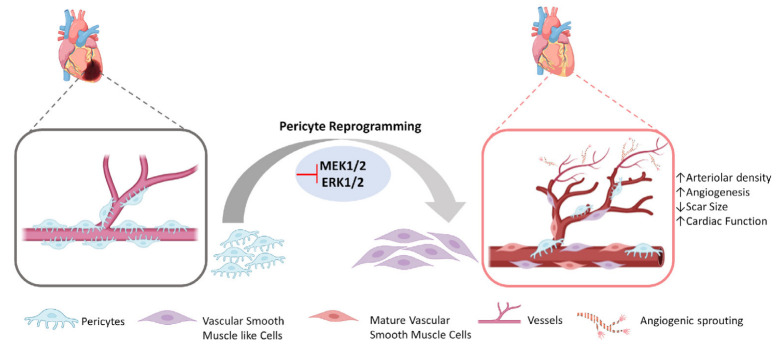
Direct reprogramming of cardiac pericytes into vascular smooth muscle like cells and their potential for angiogenesis. In the injured heart, resident cardiac pericytes can be reprogrammed into vascular smooth muscle like cells that gradually mature and contribute to angiogenesis and arteriogenesis.

A recent study has focused on deciphering the role of induced phenotypic transition of myocardial PCs in aiding cardiac neovascularization both in vitro and in vivo in a small animal model [126]. Primary PCs from human and mouse hearts, either on omission of EGF/bFGF, which signal through ERK1/2, or on introduction of MEK inhibitor PD0325901, achieved cytoskeletal protein characteristic of vascular SMCs, became more proangiogenic, and also expressed angiogenesis markers (AQP1 and CRABP2). In an in vivo mouse model, it was shown that when matrigel with embedded PCs and MEK inhibitor PD0325901 was injected into mice, αSMA+ neovessels, enhanced arteriolar density, and total vascular area could be observed. Further in situ study was performed in infarcted mouse models, and it was noted that PD0325901 treatment promoted peri-infarct vascularization, decreased scar size, and improved systolic function (Figure 3). Hence, intrinsic plasticity of PCs could be modulated to encourage reparative vascularization in the injured heart [126]. This study opens the path for other NMCs besides CFs to be reprogrammed into functional cardiac phenotypes potentially able to minimize the damage caused by cardiac injury. These authors proposed this approach for treatment of ischemic heart failure and diabetic cardiomyopathy characterized by arteriolar regression. However, this study did not focus on EC and capillary formation; for this reason, further investigations are necessary to understand whether this strategy leads to the production also of iECs.

Overall, PCs seem to offer another source of reprogrammable NMCs; however, this source still has limitations due to the lack of production of CMs, which are essential for cardiac function.

## 6. Conclusions

Direct reprogramming of NMCs is an appealing strategy worthy of further investigation. It was rendered possible by an increased understanding of cell fate determination during cardiovascular embryogenesis together with the extraordinary technological improvements related to induced cell reprogramming. High intrinsic NMC plasticity, probably due to their multi-lineage embryonic derivation, is the third important element in this strategy. A challenge for clinical translation of direct cardiac reprogramming is still the establishment of an effective cardiac reprogramming protocol for human NMCs. Although several research groups worked on reprogramming human fibroblasts into iCMs, the duration, quality, and effectiveness of human iCM reprogramming were not comparable to the mouse one. Furthermore, cardiac diseases mainly affect older subjects; for this reason, it should be kept in mind that age and inflammation might act as barriers in reprogramming NMCs into iCMs or iCPCs, because of changing NMC gene expression and activation of different signalling pathways. In conclusion, the direct reprogramming of resident NMCs into cardiac cells is a potential powerful strategy that waits for translational confirmation. Despite amazing progress having been achieved, particularly on in vitro cardiac reprogramming, progress on in vivo cardiac reprogramming for future clinical applications has been relatively slow. Not only do its safety, scalability, and therapeutic time window need to be validated, but also its full potentiality for fostering both endogenous vascularization and myocardial repair, a very prominent advantage of this innovative approach.

## Data Availability

Not applicable.
